# Bacterial endotoxin-lipopolysaccharide role in inflammatory diseases: An overview

**DOI:** 10.22038/ijbms.2025.82302.17799

**Published:** 2025

**Authors:** Priyanka Arya, Vikram Sharma, Priyanka Singh, Surabhi Thapliyal, Manu Sharma

**Affiliations:** 1 Galgotias College of Pharmacy, Greater Noida, U.P., India; 2 Banasthali Vidyapith, Department of Pharmacy, Rajasthan, India; 3 Department of Pharmacology, All India Institute of Medical Sciences, Rishikesh 249203, India

**Keywords:** Alzheimer’s, Atherosclerosis, Cancer, Cardiovascular diseases, Lipopolysaccharide, Neuroinflammation, Toll-like receptors

## Abstract

Despite advancements in antimicrobial and anti-inflammatory treatments, inflammation and its repercussions continue to pose a considerable challenge in medicine. Acute inflammation may cause life-threatening conditions like septic shock, while chronic inflammation leads to tissue degeneration and impaired function. Lipopolysaccharides (LPS), a well-known pathogenic trigger contributing to several dysfunctions, is a crucial part of the outer membrane of gr-negative bacteria. LPS are well-known for eliciting acute inflammatory responses by activating a pathogen-associated molecular pattern (PAMP), which stimulates the innate immune system and triggers local or systemic inflammatory responses. LPS also activate numerous intracellular molecules that modulate the expression of a wide range of inflammatory mediators. These mediators subsequently initiate or exacerbate various inflammatory processes. Beyond immune cells, LPS can also activate non-immune cells, leading to inflammatory reactions. These excessive inflammatory responses are often detrimental and typically result in chronic and progressive inflammatory diseases, including neurodegenerative, cardiovascular diseases, and cancer. This review delves into the mechanisms by which the bacterial endotoxin LPS contribute to multiple inflammatory diseases. These insights into LPS signaling pathways could inform the design of new treatment strategies such as TLR4, NLRP3, HMGA1, MAPK, and NF-kB inhibitors. This enables precise targeting of inflammation-related processes in disease management.

## Introduction

Lipopolysaccharides (LPS), sometimes called lipoglycan or endotoxins, are found in gr-negative bacteria’s outer membrane (1). LPS from these bacteria can trigger various inflammatory pathways, reflecting the development of chronic inflammatory diseases such as sepsis, neurodegenerative, arthritis, metabolic, and cardiovascular disease (2). This is particularly relevant at concentrations of LPS that sustain low-grade, persistent inflammation. Therefore, even at low levels within the body, LPS should be recognized as an important contributor to chronic illnesses (1). Researchers indicate that LPS triggers the neuroinflammation response in the central nervous system via modulating neuronal cells like astrocytes, microglia, cytokines, etc. These significantly contribute to neurodegenerative diseases like Parkinson’s disease, Alzheimer’s disease, and amyotrophic lateral sclerosis (2). 

 It was revealed that infection by gr-negative bacteria leads to cardiovascular diseases like myocardial infarction, atherosclerosis, heart attack, etc. Circulatory LPS function as pathogen-associated molecular patterns (PAMPs) that can trigger the innate immune system, leading to localized or systemic inflammatory responses. LPS also can activate non-immune cells, triggering inflammation (3). However, excessive inflammatory responses can be harmful. The Toll-like receptor 4 (TLR4), which detects LPS, is broadly expressed throughout the body, involving heart muscle (cardiomyocytes). This indicates that LPS can directly trigger an innate inflammatory response by oxidative stress or by influencing macrophages, monocytes, and dendritic cells (DCs). The binding proteins CD14, MD-2, and LPS cause damage to cardiomyocytes without the involvement of immune cells. As a result, there is precipitation of clinical symptoms such as dyslipidemia, atherosclerosis, myocardial infarction, and stroke (3).

Researchers reported that in a healthy individual, minimal levels of LPS, up to 1–5 μg/ml of blood, can circulate without triggering any harmful effects (4). However, a rise in LPS levels can lead to different conditions or diseases, such as metabolic and neurological diseases. LPS can bind directly to its cell surface receptor, TLR4, initiating an intracellular signaling cascade through either a MyD88-dependent or MyD88-independent pathway (5). This activation leads to downstream signaling, including the PI3K/AKT and nuclear factor-kB (NF-Kappa B) pathways. 

Numerous researches have highlighted the critical role of LPS in many human cancers. For instance, LPS has been shown to enhance cell adhesion and invasion by activating TLR4 signaling in colorectal cancer. Moreover, it has been demonstrated that LPS promote invasion and metastasis in various cancer cells (6). 

This review article highlights the role of LPS in the onset and progression of cancer, cardiovascular disease, and neurodegenerative disorders. It seeks to offer researchers and pharmacologists a deeper understanding of LPS-induced inflammatory disease pathogenesis and to inform the development of more effective therapeutic strategies.

## Methodology

Scientific data was gathered using online engines and databases such as PubMed, Scopus, Science Direct, and Google Scholar. The search included articles published between January 2000 and December 2024. Inclusion and exclusion criteria articles were selected based on their relevance to the role of bacterial endotoxin (lipopolysaccharide, LPS) in inflammatory diseases. Inclusion criteria encompassed peer-reviewed original research, systematic reviews, and experimental studies focusing on the interaction between LPS and inflammatory pathways (e.g., TLR4, MAPK, CaSR, ADRP, MCP-1, NF-κB, etc., signaling). Studies involving human subjects, animal models, or *in vitro* systems are relevant to diseases like neurodegenerative, cardiovascular diseases, and cancer. Keywords used for the search included “Lipopolysaccharide”, “Neuroinflammation”, “cardiovascular diseases”, “Cancer”, “Toll-like receptors”, “Alzheimer’s”, “Atherosclerosis”, “Astrocyte,” “Glial,” “Myocardial Infraction“, “Parkinson’s”, “Breast cancer”, Exclusion criteria non-peer-reviewed articles, studies unrelated to inflammation, keywords unrelated to the topic or publications in languages other than English were excluded. 

## Structure of lipopolysaccharide (LPS)

Gr-negative bacteria have a complex glycolipid called LPS in their outer membrane. Maintaining the structural integrity of bacteria is important; it plays a significant role in host-pathogen interactions, especially in initiating immune responses. Three components comprise the LPS molecule: O antigen repeats, core polysaccharides, and lipid A. The outer leaflet of the outer membrane contains lipid A, which makes up the hydrophobic portion of LPS. On the other hand, the bacterial cell surface contains core polysaccharides and O antigen (7).

***Lipid A***

Lipid A is a critical component and structural element for the bacterium and host. The hydrophobic segment of the LPS molecule secures LPS to the gr-negative bacteria’s outer membrane. It comprises a disaccharide backbone formed by two glucosamine units connected through a β (1→6) glycosidic linkage**).** The phosphorylation of glucosamines at positions 1 and 4’ and acylation at positions 2, 3, 2’, and 3’ in *Salmonella* and *Escherichia coli* results in the molecule having a negative charge. The negatively charged phosphates are essential for interacting with divalent cations such as Mg²⁺ and Ca²⁺, which help stabilize the bacterial outer membrane. The phosphorylation pattern of Lipid A is vital for its biological function (8)**.** For Lipid A to engage with the host’s innate immune system, phosphate groups at positions one and four are essential. The TLR4-MD-2 receptor complex on the surface of immune cells is particularly engaged by these phosphate groups, starting a signal transduction cascade that produces proinflammatory cytokines. Certain bacteria add extra moieties to Lipid A’s phosphate groups, like phosphoethanolamine or 4-amino-4-deoxy-L-arabinose (9). These changes may lessen Lipid A’s net negative charge, making it more resistant to cationic antimicrobial peptides and less likely to trigger the host immune response. Lipid A’s structure is inconsistent across all gr-negative bacteria; it varies between species and even within a single species under different environmental conditions. For instance, in *E. coli*, Lipid A typically has six fatty acid chains and is hexa-acylated. This arrangement is highly immunostimulatory and can induce potent endotoxic effects. In *Salmonella*, Lipid A can be hexa-acylated, similar to *E. coli*. However, it may be modified to a penta-acylated form under specific conditions, which can change its immunogenic properties (10). The main element in LPS that causes the endotoxic effects is lipid A. 

When bacteria produce LPS during infection, or after bacterial lysis, Lipid A binds itself to the TLR4 receptor on immune cells, including dendritic cells and macrophages. Tnf-α, IL1, and IL6 are released due to this interaction, which causes inflammation. Although this immune response is essential for managing bacterial infections, high doses of LPS can overactivate it and cause septic shock, a potentially fatal illness marked by extensive inflammation, blood clotting, and organ failure (11).

***Core oligosaccharide***

The core oligosaccharide of LPS is a more conserved component than the variable O-antigen, providing structural integrity and influencing interactions with the host immune system (12). It consists of two main parts: 

1. The inner core: contains conserved sugars like L-glycero-D-manno-heptose and 3-deoxy-D-manno-octulosonic acid (Kdo). Kdo is most important for linking the lipid A’s core oligosaccharide, ensuring the structural stability of the LPS molecule (13).

2. Outer core: It comprises variable sugars like glucose, galactose, and N-acetylglucosamine. This variability affects bacterial serotypes and their interactions with the host immune system (14). 

While the inner core remains relatively uniform across gr-negative bacteria, the outer core’s diversity can influence the pathogen’s ability to evade immune recognition. For example*, E. coli *strains show only five distinct core types (R1 to R4 and K-12) despite having over 160 O-antigen variations (12). Similarly,* Salmonella *has a single core type, while *Proteus* and *Citrobacter* serotypes exhibit more diversity (15).

The core oligosaccharide acts as a pathogen-associated molecular pattern (PAMP), identified by host immune responses, including inflammation (12). However, the pathogen can modify their core oligosaccharides to reduce detection by the immune system, enabling immune evasion. For example, structural variations in the outer core may alter the intensity or specificity of immune activation, impacting the host’s ability to fight infections (14). This balance between conservation and variability in the cire oligosaccharide highlights its dual role in maintaining LPS stability and mediating host-pathogen interactions, linking it directly to the immune responses and pathogenicity associated with gr-negative bacteria. 

***O-Antigen***

The O-antigen, the outermost portion of LPS, is a highly versatile polysaccharide necessary for immune evasion and bacterial virulence. It comprises repeating sugar subunits that differ widely among bacterial strains in sequence, linkage, and substituents. This variability allows gr-negative bacteria to adapt to diverse environments and evade host immune recognition. For example, strains like *E. coli* O8 and O9 produce O-antigens made entirely of D-mannose, demonstrating the structural diversity among serotypes (16-17).

The O-antigen is synthesized using enzymes encoded by the *rfb* gene cluster, including glycosyltransferases, polymerases (e.g., Wzy and Wzz), and transport proteins. After synthesis on the inner membrane, the O-antigen is associated with lipid A-core oligosaccharide by WaaL, forming mature LPS. Wzz regulates O-antigen chain length, ensuring optimal immune evasion and bacterial survival balance, while Wzy polymerizes the repeating units. The absence of O-antigen, as seen in “rough” mutants, increases bacterial susceptibility to hydrophobic antibiotics and immune defense (18). 

Functionally, the O-antigen acts as a shield against host defenses. Its hydrophilic nature reduces bacterial surface hydrophobicity, limiting antibiotic recognition and resisting phagocytosis or complement-mediated killing. This is particularly relevant in LPS-related diseases like septic shock, where LPS triggers excessive inflammatory responses, and chronic infections, where O-antigen variability contributes to immune persistence (19).

## LPS role in cardiovascular diseases

Cardiovascular disease (CVD) is characterized by both high disability and mortality rates. As its incidence continues to rise globally, it represents a significant risk to human life and health (20). Initially, it was thought that cardiovascular disease was only the cause of dyslipidemia, which leads to myocardial infarction, heart attack, and atherosclerosis. Later, researchers learned that there is a strong bridge between inflammation and CVDs. Increasing evidence suggests its pathophysiology involves multiple pathways, with inflammation playing a central role in its onset and progression (21). Vascular inflammation, in particular, is recognized as an early indicator and a common underlying pathophysiological factor in various cardiovascular conditions (22).

Endothelial cells are essential for maintaining vascular homeostasis since they are the primary interface between blood and vascular tissue (23). When exposed to harmful factors such as pathogens, bacteria, hypoxia, endotoxins, oxidized low-density lipoprotein, and inflammatory cytokines, these cells can shift from dormant to active. Endothelial cells produce a range of proinflammatory cytokines, enzymes, chemokines, and adhesion molecules when they are activated. These inflammatory cytokines cause endothelial cells to become proinflammatory by increasing the inflammatory proteins and activating several signaling pathways (24). In this inflammatory condition, endothelial cells can incite inflammation within the vessel wall, disrupting its normal function. This leads to impaired barrier function, heightened permeability, and intensified inflammation, all of which contribute to the development of cardiovascular diseases such as dyslipidemia, atherosclerosis, myocardial infarction, and stroke (25).

## LPS’s role in cardiac dysfunction

LPS, a potent gr-negative bacterium, initiates the release of various endogenous mediators, leading to hypotension, multi-organ failure, and potentially fatal outcomes from sepsis and septic shock. LPS impairs cardiac function, mitochondrial functions, cardiac contractility, oxidative stress, and vascular flexibility, contributing to the onset of hypotension during sepsis. Several cytokines released in response to LPS are known to suppress cardiac function and mediate many of the vascular and lethal effects associated with LPS (26).

**Figure 1:**
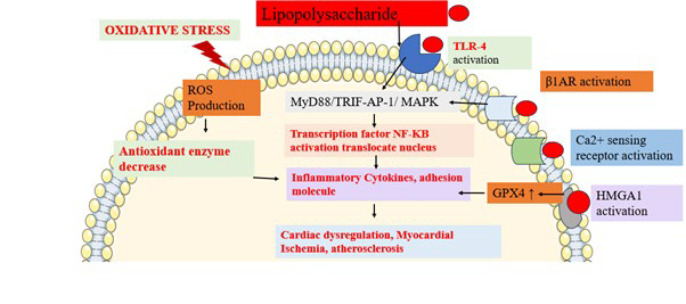
Mechanistic insight of LPS in cardiovascular diseases

The heart is a vital organ highly vulnerable to sepsis-induced damage, with myocardial dysfunction being a serious complication resulting from the excessive generation of free radicals (27). Nuclear factor erythoid2-related factor 2 ((Nerf2). is a transcription factor that can be stimulated via oxidative stress. This activation subsequently increases the mRNA expression of Nrf2 target genes, such as hemeoxygenase 1 (HO1). HO1 protects against hypoxia/reoxygenation-induced heart damage (28). Investigators reveal that modulation of the Nrf2/HO1 signaling pathway is involved in cardiomyopathy induced by LPS (Figure 1). In 2023, Sun and colleagues conducted research showing that when H9c2 cardiomyocyte cells were incubated with LPS (10 µg/ml) for 24 hr at 37 °C, there was a noticeable reduction in the levels of expression of Nrf2 and cellular HO1 in the LPS-treated cells (29). Moreover, they observed a notable rise in NLRP3, proIL1β, and ILs1β proteins. This finding suggests that LPS induces oxidative stress and inflammatory responses, which may contribute to the development of cardiomyopathy (26, 30).

Cowan *et al*. 2001 demonstrated *in vitro* studies in which they evaluated the bacterial LPS internalization to trigger endotoxin-dependent signaling in cardiac myocytes. They discovered that primary cardiomyocyte cultures treated with fluorescently labeled LPS perfused cardiac preparations with the cell line of RAW264.7 macrophage. Spectrophotometry and confocal microscopy demonstrated that cardiomyocyte cells and Langendorff-perfused hearts quickly absorbed LPS. Although macrophages were also seen to absorb LPS, only a portion of these cells internalized the endotoxin to an extent similar to cardiomyocytes. Additionally, they used organelle- or structure-specific fluorophores for colocalization experiments, which showed that LPS accumulated in lysosomes, sarcomeres, and the Golgi apparatus. Using gold-labeled LPS and transmission electron microscopy, its intracellular location was further confirmed in cardiomyocytes. The lipopolysaccharide (LPS) complex was determined to depend on endosomal trafficking. In both cardiomyocytes and entire hearts, its uptake was inhibited by a microfilament rearrangement inhibitor (31).

Additionally, they noted that LPS treatment activated extracellular signal-regulated kinase (ERK) proteins and NF-Kappa B, resulting in the production of Tnf-α and the expression of inducible nitric oxide synthase. The researchers concluded that endotoxin-related signalling in cardiomyocytes is activated by LPS treatment, which may lead to cardiovascular disease (31). 

TLR4, the innate pattern recognition receptor for LPS, is broadly expressed throughout the body, including in cardiomyocytes (32). As a result, LPS can trigger an innate inflammatory response in cardiomyocytes without the need for involvement from immune cells (33). This may be the reason cardiac dysfunctions are commonly observed in humans with sepsis and in animals administered with LPS (34) (Figure 1).

Investigators reported that acute infections like sepsis resulted in cardiomyopathy. Yücel *et al*., 2017 reported that LPS induces an inflammatory response, which leads to alteration in the electrophysiology of human-induced pluripotent stem cell-derived cardiomyocytes. Human induced pluripotent stem cell-derived cardiomyocytes (hiPSC-CMs) were exposed to LPS at various concentrations for differing durations. It was observed that proinflammatory and chemotactic cytokines (Tnf-α, IL1ß, CCL-2, IL6, IL9, and CCL5) were more highly expressed after six hours of LPS treatment, while anti-inflammatory factors (IL10 and IL6) were more highly expressed after 48 hr in cardiac myocytes. They concluded that LPS damages the cardiomyocyte cells (35).

Intrinsic cardiac adrenergic (ICA) cells, which produce catecholamines by expressing enzymes like tyrosine hydroxylase (TH) and dopamine β hydroxylase (DBH), have been identified as a significant source of intrinsic cardiac catecholamines in the mammalian heart. Emerging research indicated that the ICA system is crucial in regulating heart development, providing inotropic support after heart transplantation, and offering cardioprotection during ischemia/reperfusion (36). Researchers reported that LPS triggers immune cell pathogen-associated molecule signaling TLR4-MyD88/TRIF-AP-1 and stimulates the release of norepinephrine by activating ICA Cells, which results in the release of various inflammatory cytokines and leads to cardiomyopathy (37). Yang *et al*., 2021 reported that LPS induces cardiomyopathy in neonatal Sprague-Dawley rats ICA cells. LPS injection treatment leads to sepsis-induced myocardial dysfunction (SIMD) via binding to TLR4 on immune and cardiac cells. This interaction triggers the stimulation of NF-Kappa B and mitogen-activated protein kinase (MAPK) signaling pathways, leading to a surge in proinflammatory cytokines like Tnf-α, IL1β, and IL6, directly contributing to cardiac depression. Additionally, norepinephrine (NE), which has been linked to SIMD, can activate β1-adrenergic receptors, promoting the phosphorylation of calcium/calmodulin-dependent protein kinase II and IκBα, as well as increasing Tnf-α expression, all of which contribute to cardiomyocyte dysfunction. Researchers concluded that inhibiting β1-adrenergic receptors and ICA cell release may be a potential therapeutic target to prevent cardiomyopathy. Researchers have also found that LPS activates the calcium sensing receptor, a member of the family C G-protein coupled receptors, contributing to cardiac dysregulation (38). Wang and coworkers in 2013 demonstrated that when cultured neonatal rat cardiomyocytes were exposed to LPS, there was an increase in cardiomyocyte apoptosis, MDA and LDH levels, Tnf-α and IL6 release, as well as elevated CaSR protein expression (Figure 1) (39).

## LPS’s role in atherosclerosis

Chronic degenerative artery disease, or atherosclerosis, is one of the world’s major causes of fertility problems in humans. Researchers have shown that inflammation contributes to the etiopathogenesis of atherosclerosis (40). One of the main causes of arterial inflammation during atherosclerosis is inflammation, which includes elements of harmful bacteria, especially LPS (41).

LPS has been shown to cause inflammatory reactions in human vascular endothelial cells. 

Research involving human tissue and primary cell cultures has demonstrated that LPS stimulates the expression of neuraminidase-1 in monocytes, an enzyme associated with the advancement of atherosclerosis (42). This enzyme is involved in a positive feedback loop that enhances the expression of proinflammatory and pro-atherogenic cytokines. Additionally, administering subclinical doses of LPS to mice results in decreased levels of interleukin-1 receptor-associated kinase M (IRAK-M) and elevated expression of miR-24, a precursor microRNA in monocytes, which play a vital role in mitigating inflammation and preserving homeostatic tolerance. This system’s disruption contributes to sustained, unresolved low-grade inflammation (Figure 1) (43).

LPS treatment of human aortic adventitial fibroblast cells led to increased lipid deposition by up-regulating adipose differentiation-related protein (ADRP). It also enhanced monocyte chemoattractant protein (MCP-1) release from these fibroblasts. Additionally, LPS increased the expression of TLR4 and NF-Kappa B, which led to a greater release of proinflammatory cytokines, artery constriction, and arterial stiffness (44). The generation of reactive oxygen species (ROS) has been intimately associated with the genesis and progression of atherosclerosis (45). Investigators reveal that exposure of LPS to endothelium cells up-regulates oxidative stress (46) (Figure 1).

LPS can strongly stimulate the generation of ROS in phagocytes via stimulating nicotinamide adenine dinucleotide phosphate (NADPH) oxidase. It also promotes O2− production by facilitating the phosphorylation of p47phox and its movement to the plasma membrane (47, 48). In blood arteries, this enzyme is present in endothelial cells, vascular smooth muscle cells, and fibroblasts, contributing to the release of inflammatory cytokines and the growth and programmed death of smooth muscle cells. This suggests a significant role for NADPH oxidase in the development of atherosclerosis (49).

## LPS role in myocardial infarction

There is a close relationship between inflammation and cardiac tissue damage. Investigators discovered that LPS affects the high Mobility Group A1 (HMGA1) proteins linked to cardiomyocytes by regulating gene expression and chromatin structure. In cardiomyocytes, HMGA1 influences the transcription of genes critical for cell growth, differentiation, and stress responses (Figure 1) (50). Its dysregulation can lead to maladaptive cardiac remodeling, hypertrophy, and heart failure. HMGA1 has also been implicated in protecting cardiomyocytes from stress-induced damage via regulating signaling pathways involved in survival and apoptosis (51). Therefore, it is crucial to preserve cardiomyocyte function and structural integrity, especially under pathological conditions like myocardial infarction and heart disease. H9c2 rat cardiomyocytes were treated with LPS for 12 hr. HMGA1 overexpressed in inflamed murine hearts and LPS-stimulated H9c2 cardiomyocytes. Overexpression of HMGA1, cyclooxygenase COX-2, and increased inflammatory cytokine release worsened cardiac dysfunction, heightened inflammation, and increased cell apoptosis after LPS treatment in both *in vivo* and *in vitro* experiments (52). Researchers also reported that LPS induces myocardial injury by activating ferroptosis, which increases Glutathione peroxidase 4 (GPX4) secretion and increases transcription of lipid peroxidation-related mRNAs, which leads to myocardial damage. This evidence suggests that LPS is involved in myocardial infarction (53) (Figure 1).

## Role of LPS in cancer

**Figure 2:**
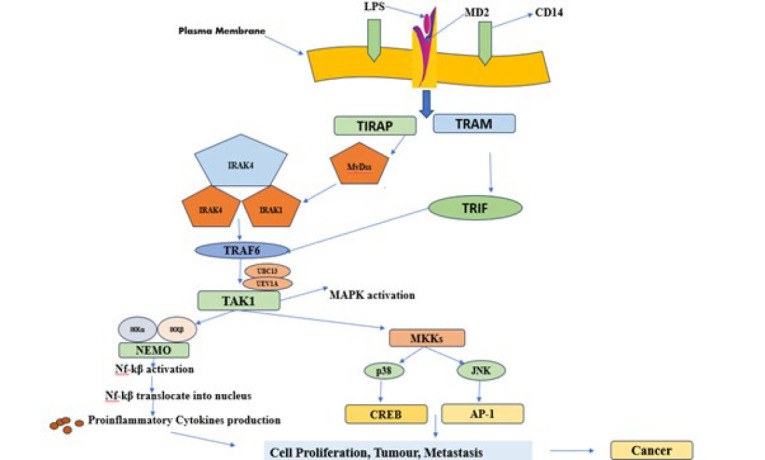
The mechanistic insight of LPS in cancer

Cancer is a broad category of diseases that can develop in almost every organ or tissue in the body. It is marked by the unchecked proliferation of atypical cells, which can infiltrate nearby tissues and potentially metastasize to other organs. This process is called metastasis, which leads to the majority of deaths. Many epidemiologic and occupational health studies are witness to the significance of exposure to known or suspected carcinogens as well as lifestyle factors in the development of cancer. Indeed, 15–20% of cancer cases are thought to be caused by infectious organisms. The connection between the immune system and bacteria arises from the presence of microbiota in the human gut since birth. Live bacteria or bacterial components frequently trigger innate immune responses in host-pathogen interactions. Due to this stimulation, immune cells like monocytes and macrophages move toward tumors (54). The innate immune system is the first line of defense against infections or tissue damage. Identifying pathogens or endogenous ligands is driven by the generation of pathogen-associated molecular patterns (PAMP) or damage-associated molecular patterns (DAMP). These patterns are produced in response to infection, tissue damage, or necrosis, enabling the immune system to identify potential threats. PAMP and DAMP identification during the innate immune response is a key function of TLRs, a class of pattern recognition receptors (PRRs) (55). TLRs perform their functions by triggering the generation of inflammatory cytokines and antimicrobial activity. They are also essential for tissue regeneration and repair (56). Bacteria contribute to cancer development primarily through two mechanisms: producing carcinogenic compounds and inducing chronic inflammation. These processes can promote the conditions necessary for tumor growth and progression (57)**.** Numerous bacteria have been linked to cancer, including Mycoplasma hyorhinis, whose p37 protein has been shown to enhance tumor invasiveness in humans. These bacterial connections highlight the potential role of microbial factors in cancer progression (58). Porphyromonas gingivalis, Granulicatella adjacens (associated with systemic inflammation), along with antibodies against various oral bacteria, have been frequently detected in patients with pancreatic cancer. Helicobacter pylori is the primary causative agent of stomach ulcers and gastric cancer (59)**.** Research has shown that *Fusobacterium nucleatum* influences the infiltration of lymphocytes into tumors and contributes to carcinogenesis (60). Additionally, it impairs the ability of Natural Killer cells and tumor-infiltrating T cells to kill cancer cells while also promoting resistance to chemotherapy in colon and breast cancers (61, 62) (Figure 2).

LPS, a key structural element in the gr-negative bacteria’s outer membrane, can enter the bloodstream and subsequently trigger systemic inflammation and sepsis. A healthy individual’s bloodstream can contain tiny amounts of LPS (1-5 pg/ml) without experiencing any significant adverse effects. Following a bacterial infection, the concentration of lipopolysaccharide in the blood can rise significantly, reaching levels as high as 300 pg/ml in sepsis and septic shock cases, or even 1 to 2 μg/ml, considered a lethal dose (63)**. **TLR4 on the surface of immune and cancer cells recognizes LPS. Pancreatic, liver, breast, gastric, and colorectal cancers are among the cancer types where LPS acts as the primary activator of TLR4. Upon stimulation by LPS and activation of TLR, macrophages experience various biochemical and metabolic alterations. The interaction between LPS and TLR4 triggers a cascade of downstream signaling pathways, primarily resulting in the activation of NF-Kappa B. NF-Kappa B is a transcription factor that controls the expression of various proinflammatory cytokines, such as IL6, IL1β, and Tnf-α. These cytokines foster a proinflammatory environment that promotes cancer cell growth, survival, and invasion (64). directly linked to the decreased levels of TCA cycle intermediates and the increased levels of aerobic glycolysis, as seen in the metabolomic analysis of LPS-stimulated macrophages. Large amounts of metabolites like lactate and succinate are secreted due to the fragmented nature of the TCA cycle. The cytotoxic effect of CD8 + CTLs and the motility of activated T cells *in vitro* are inhibited by the increased generation of lactate in LPS-stimulated macrophages. Macrophages produce ARG1 and VEGF in response to high lactate, which activates HIF-1α and MAPK (65). By causing angiogenesis and arginase depletion, VEGF and ARGI encourage tumor growth. As a proinflammatory metabolite, succinate raises ROS generation and suppresses prolyl hydroxylase activity, stabilizing HIF-1α. LPS is a potent inflammatory mediator that not only encourages the growth of tumors (66) but also promotes cancer dissemination via β1 integrin-mediated cell adhesion and contacts between cancer and endothelial cells triggered by monocytes (67). 

## LPS role in colon cancer

*In vitro* experiments and preclinical models have demonstrated that LPS promotes CRC cell adhesion and metastasis through the TLR4 inflammatory signaling pathway (68). Additionally, LPS enhances VEGF-C release, which stimulates lymphangiogenesis and cell motility through the TLR4- NF-Kappa B /JNK signaling pathway. Furthermore, it causes caspase-1 to become activated via NF-Kappa B, resulting in the increased expression of Snail and HK3, which is contingent upon the activation of caspase-1 in colorectal cancer (Figure 2) (69). In *in vitro* and preclinical studies, LPS also promoted colon cancer cell invasion and metastasis by triggering the NF-κB NF-Kappa B signaling cascade through the SDF-1α/CXCR4 axis (65)**. **

## LPS role in pancreatic cancer

There is accumulating evidence that LPS can activate Programmed Cell Death Ligand 1 (PD-L1) expression and improve immune escape in pancreatic cancer by activating the TLR4/MyD88/ NF-Kappa B pathway and the P13K/Akt/mTOR signaling pathway. This establishes a connection between inflammation and the advancement of cancer (70-72) (Figure 2). 

## LPS role in breast cancer

LPS is also associated with breast cancer via various signaling pathways. Researchers reported that LPS improves the response of ER+ BC and TNBC to IAP antagonist therapy. TLR-4 and MyD88 achieve this impact-mediated Tnf-α production, which induces cell death. Combining SM-164 with systemic Tnf-α or LPS injection may prove to be a successful treatment for progressive breast tumors, even those that have spread to other organs. LPS up-regulates S100A7 expression in breast cancer cells, which in turn regulates TLR4 and RAGE expression, influencing breast tumorigenesis (73)**. **Li *et al*. (2017) state that LPS-induced TLR4 activation can cause undesirable phenotypes in breast cancer cells. The cells were exposed to lipopolysaccharide (LPS), which induced the Wnt/b-catenin signaling pathway, which is constitutively activated in several cancer types. A transcription factor called b-catenin activates the various genes linked to cancer spread. It is usually strictly controlled by a destruction complex that breaks down b-catenin before it can enter the nucleus. Nevertheless, the destruction complex is deactivated in the presence of Wnt signaling, enabling b-catenin to activate genes linked to metastasis (Figure 2) (66). LPS-induced systemic inflammation increases VEGF synthesis and pulmonary angiogenesis in mice, mainly via the PGE2/EP2 receptor pathway, encouraging lung metastasis of breast cancer cells (74). When monocytes treated with LPS produced thrombin, it improved the arrest of breast cancer cells by attaching them to active endothelium cells (75). 

## LPS’s role in gastric cancer

Gastric cancer development has also been linked to LPS through its autophagy regulation, cell proliferation, and the epithelial-mesenchymal transition (EMT) (76). Specifically, it has been discovered that *Helicobacter pylori* LPS activates the transcription 3 (STAT3) signaling pathways as well as the NF-Kappa B and signal transducers (77). By activating transforming growth factor β (TGF-β) signaling pathways, NF-Kappa B drives PD-L1 and encourages EMT. This activation is crucial to the development of cancer (Figure 2) (78). In a clinical study conducted by and coworkers involving 198 gastric cancer patients who underwent surgery, the group with lipopolysaccharide positivity exhibited higher levels of cancer stromal TGFB1 expression and increased PD-L1 expression in cancer cells. A strong correlation was identified between lipopolysaccharide positivity and increased Wnt3a signaling, coupled with diminished E-cadherin expression (79). 

## LPS role in hepatocellular carcinoma (HCC)

HCC typically develops in an environment of persistent liver injury, often as a consequence of liver fibrosis. In cases of chronic liver injury, hepatic progenitor cells (HPCs) can differentiate in both hepatocytes and cholangiocytes in chronic liver damage. These cells are also believed to play a role in developing tumor-initiating cells and myofibroblasts in cancerous circumstances (80). Wang *et al*. discovered that overexpression of TLR4 in human HCC tissues and cell lines of HCC was correlated with levels of Ki-67. LPS-induced TLR4 signaling influenced the NF-Kappa B, MAPK, ERK, and JNK pathways, which in turn enhanced the survival and growth of cancer cells (81). This signaling pathway also enhanced cell proliferation by influencing the migration of Bax into the mitochondria. Furthermore, LPS-induced cytotoxicity was mediated by the stimulation of the NF-Kappa B and p38 pathways, and their suppression led to enhanced cell proliferation in HCC cells (Figure 2) (82).

## LPS role in neuroinflammation

**Figure 3:**
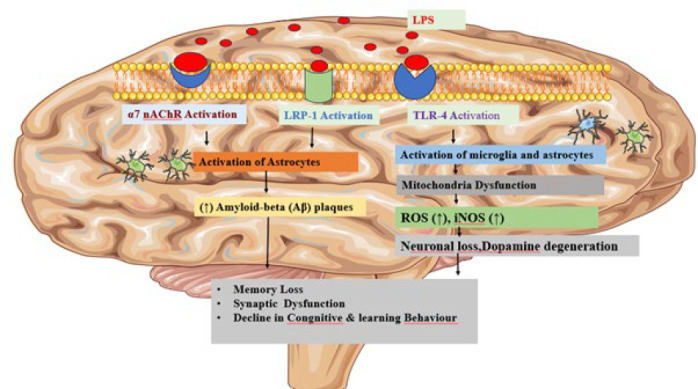
The mechanistic insight of LPS in neuroinflammation

The nerve tissue inflammation, which frequently affects the central nervous system (CNS), is known as neuroinflammation. It is mainly driven by the activation of immune cells in the CNS, such as microglia and astrocytes (83). This process can be initiated by stimuli like LPS, which can cause tissue damage and contribute to the development and progression of neurodegenerative diseases such as amyotrophic lateral sclerosis ((ALS), multiple sclerosis, Parkinson’s disease, and Alzheimer’s disease (84). LPS was initially recognized as a ligand for TLR-4. The CNS’s microglia are the primary site of TLR-4 expression, and once activated, it includes the synthesis of proinflammatory cytokines such as Tnf- α, IL1β, prostaglandin E_2_(PGE_2_), and NO (85). LPS are potent activators of peripheral immune cells, including macrophages and monocytes, as well as CNS glial cells, such as microglia and astrocytes (Figure 3). These cytokines serve as pivotal mediators in the neuro-inflammatory process. More significantly, TLR-4 facilitates extensive neuronal cell death. Many of these symptoms are considered to closely resemble the clinically relevant symptoms of neurodegenerative diseases in humans (86). The development of several neurodegenerative illnesses, including multiple sclerosis, Parkinson’s disease, and Alzheimer’s disease, is linked to prolonged or severe LPS-induced neuroinflammation (86).

## LPS’s role in parkinson’s

Parkinson’s disease (PD) is marked by neuropathology that includes the degeneration of dopaminergic neurons within the substantia nigra, leading to the loss of axonal connections in the striatum and dysfunction of the dopaminergic system (87). Dopaminergic impairment results in the characteristic motor symptoms of PD, including tremors, rigidity, bradykinesia, and abnormalities in posture and gait (88). PD is characterized by Lewy bodies, which are cytoplasmic inclusions mostly made of deposits of the protein α-synuclein (89). LPS has been widely used as a glial activator to induce inflammatory neurodegeneration in dopaminergic neurons. LPS triggers rapid microglial activation within hours, as shown by the morphological changes in OX-42-positive microglia, the up-regulation of proinflammatory cytokines, and the production of free radicals (96). Degeneration of dopaminergic neurons in the substantia nigra pars compacta (SNpc) was found one week following the LPS injection. In contrast to the swift activation of microglia, astroglial activation occurred more slowly, becoming significant about one week after LPS administration (Figure 3) (90).

Microglia-driven neuroinflammation is crucial in the neurodegenerative progression of PD. Administering drugs that alleviate the effects mediated by microglia supported the role of inflammation. Systemically administering dexamethasone for eight or fifteen days avoided the reduction in TH activity and TH immunostaining brought on by intraneural LPS injection. This suggests a reduction in dopamine dysfunction alongside a decrease in microglia activation (91). These studies were the first to substantiate the idea that microglia-mediated neuroinflammation contributes significantly to the neurodegenerative process of PD.

Mitochondrial malfunction is also linked to the death of neurons during development (92). The mitochondrial respiratory chain was altered by intrastriatal LPS injection. Elevated concentrations of oxidative stress markers, including 3 nitrotyrosine, 4 hydroxynonenal, and protein carbonyls, indicated these alterations. Furthermore, structural alterations in the mitochondrial cristae were noted, which resulted in striatal neuronal loss and energy dysfunction. Hunter and associates discovered that l-N6-(l-iminoethyl)-lysine inhibited inducible nitric oxide synthase (iNOS), which decreased dopaminergic degeneration and mitochondrial damage brought on by LPS injection into the substantia nigra (SN). This suggests that mitochondrial dysfunction is linked to nitric oxide (NO) generated from iNOS. The p38 MAP kinase mediates the activation of iNOS, inhibiting p38 decreased cell death (Figure 3) (93).

## LPS role in alzheimer’s

Alzheimer’s disease (AD) is linked to neuropathological alterations, such as the development of tau aggregates that appear as intraneuronal neurofibrillary tangles, intraneuronal neurofibrillary tangles, and the occurrence of extracellular amyloid beta (Aβ) plaques. Studies have shown that activated microglia are found in the brain’s areas with Aβ deposits and loss of neurons, ultimately leading to memory impairment memory (94).

According to published research, prolonged administration of LPS results in impaired spatial memory in SD rats (95). Furthermore, it has been proved that LPS-induced inflammation encourages amyloid deposition *in vivo* (96). Repeated systemic LPS injections (three or seven times) led to the accumulation of Aβ 1-42 in the hippocampus and cerebral cortex of ICR albino mice. This accumulation was due to an increment in beta and gamma-secretase activity and astrocyte activation, which occurred alongside cognitive impairment (97). Additionally, it has been shown that inflammation triggered by LPS can accelerate the progression of multiple neurodegenerative processes (98). However, immune system stimulation with low doses of LPS can activate cells that help resolve neurodegenerative pathology (99) (Figure 3).

There is a connection between AD and the low-density lipoprotein receptor family member lipoprotein receptor-related protein-1, which has been shown to be involved in the metabolism of amyloid-beta (Aβ) (100). Certain studies have indicated that LPS can cause amyloid-beta (Aβ) transport dysfunction at the blood-brain barrier (BBB) via mechanisms reliant on LRP-1. LPS injections administered repeatedly intraperitoneally (IP) changed the way Aβ was transported across the BBB by increasing its influx and decreasing its efflux. Additionally, LPS was found to increase the expression of neuronal LRP-1, potentially leading to greater production and accumulation of Aβ in the brain (101). On the other hand, LPS-induced inflammation might be a valuable tool for enhancing drug delivery across the BBB. For instance, according to Barton and associates (2018), LPS improved the brain’s ability to receive small substances like thioflavin S by disrupting the blood-brain barrier in 5XFAD animals. The neuroinflammatory process may have two roles in the pathogenesis of AD by rupturing the blood-brain barrier, affecting the brain’s ability to eliminate Aβ, and maybe making pharmacological treatment easier (102).

Elevated levels of amyloid-beta (Aβ) triggered by LPS can promote the formation of neurofibrillary tangles. A single LPS injection has been shown to elevate soluble Aβ and phosphorylated tau levels in the brains of experimental animal models (103,104). Additionally, it was shown that LPS administration in the experimental model reduced the levels of the α7 nicotinic acetylcholine receptor in the brain, indicating an additional way that LPS causes neuroinflammation and cognitive impairment in AD models (Figure 3) (105). Animal models are necessary for studying tau hyperphosphorylation and Aβ accumulation, and there is compelling evidence that neuroinflammation is a key factor in neurodegeneration. In conclusion, LPS injection models are thought to replicate loss of memory and the neuropathology characteristic of AD, thereby elucidating the role of neuroinflammation in the disease’s progression (105).

## Conclusion

Lipopolysaccharide (LPS) is increasingly linked to inflammatory diseases and is known to contribute to various pathological conditions. Several mechanisms have been proposed to explain the relationship between the etiopathogenesis of chronic inflammatory illnesses and LPS-induced signaling and immunological dysregulation. LPS may stimulate and accelerate different signaling pathways MyD88/TRIF- AP-1/ MAPK, NF-Kappa B, β1AR, Ca^2+^ sensing receptor, HMGA1, trigger oxidative stress, which results in the release of multiple inflammatory cytokines, adhesion molecules, ROS, lead to impaired blood flow cardiac dysfunction, atherosclerosis, and myocardial infarction. Accumulated data also supports that LPS triggers TLR4, while CD14 and MD2 serve as auxiliary proteins for binding LPS to TLR-4.

TLR-4 dimerizes when it binds to a ligand, which then attracts downstream adaptor proteins like TRIF/TRAM and MyD88 to start an inflammatory response. After activation, MyD88 activates the IRAK4, TRAF6, TAK1, and IKK complexes. Following this, these two pathways converge at NF-Kappa B. IκB, which is broken down by proteasomes, renders the cytoplasmic NF-Kappa B complex dormant, allowing NF-Kappa B to translocate into the nucleus. TAK1 phosphorylates MAPKs and activates NF-Kappa B, intensifying the inflammatory response and promoting cancer growth. Researchers provide evidence that LPS is a potent inducer for neuroinflammation by activation of astrocyte, microglia-cells, and TLR4, α7 nAChR, LRP-1 receptors, Aβ accumulation, and tau phosphorylation, which leads to neuronal loss, synaptic dysfunction, memory loss, decline in cognitive behaviors results in PD and Alzheimer’s disease. LPS are utilized to investigate the inflammatory response and its effects in both *in vitro* and *in vivo* research. This research offers valuable insights into the underlying pathology. It paves the way for developing new treatment approaches for various medical conditions, including cardiovascular and neurological conditions and cancers.

LPS: lipopolysaccharide, MD2: myeloid differentiation factor 2, TLR4: Toll like receptor-4, MyD88: Myeloid differentiation primary response 88, TRIF: Toll/IL-1R-domain-containing adaptor-inducing IFN-β, MAPK: mitogen-activated protein three kinases, NF-κB: Nuclear factor kappa- B cells, ROS: Reactive Oxygen Species, HMGA1: High Mobility Group A1, β1AR: β 1 -adrenoceptor.

TLR4 is activated by LPS, whereas CD14 and MD2 act as accessory proteins for LPS/TLR4 binding. Upon ligand binding, TLR4 dimerizes, and recruits downstream adaptor molecules such as MyD88 and TRIF/TRAM to mount an inflammatory response. The activated MyD88 then activates IRAK4, TRAF6, TAK1, and IKK complexes. After this, both these pathways converge at NF-κB. The cytoplasmic NF-κB complex is maintained in the inactive state by IκB, which is in turn degraded by proteasomes, resulting in the translocation of NF-κB into the nucleus. Besides activating NF-κB, TAK1 also phosphorylates MAPKs to further reinforce the inflammatory response and results carcinoma.

LPS: lipopolysaccharide, MD2: myeloid differentiation factor 2, TLR4: Toll like receptor-4, CD14: cluster of differentiation 14, MyD88: Myeloid differentiation primary response 88, TRIF: Toll/IL-1R-domain-containing adaptor-inducing IFN-β, TRAM: TRIF-related adaptor molecule, TAK-1: Transforming growth factor-β activated kinase 1, MAPK: mitogen-activated protein three kinases, IRAK: Interleukin-1 receptor associated kinase, NF-κB: Nuclear factor kappa-light-chain-enhancer of activated B cells.

TLR4 is activated by LPS by making complex LPS and TLR4 which leads to stimulate the microglia and astrocytes cell which results mitochondrial dysregulation and produce reactive oxygen species and leads neuronal loss. LPS activates the LRP-1 as well as α7 nAChR which results activation of astrocyte cell and rise in amyloid beta level which leads to memory loss, synaptic neuronal loss, impair cognitive behavior.

TLR4: Toll like receptor-4, LRP-1: low-density lipoprotein receptor-related protein 1; as α7 nAChR: α7 nicotinic acetylcholine receptor, ROS: reactive oxygen species; iNOS: inducible nitric oxide synthase.

## Declaration

We have not used any AI tools or technologies to prepare this manuscript.
